# Somatic diseases in patients with schizophrenia in general practice: their prevalence and health care

**DOI:** 10.1186/1471-2296-10-32

**Published:** 2009-05-09

**Authors:** Marian JT Oud, Betty Meyboom-de Jong

**Affiliations:** 1 Department of General Practice, University Medical Centre Groningen, University of Groningen, Antonius Deusinglaan 1, 9713AV Groningen, The Netherlands

## Abstract

**Background:**

Schizophrenia patients frequently develop somatic co-morbidity. Core tasks for GPs are the prevention and diagnosis of somatic diseases and the provision of care for patients with chronic diseases. Schizophrenia patients experience difficulties in recognizing and coping with their physical problems; however GPs have neither specific management policies nor guidelines for the diagnosis and treatment of somatic co-morbidity in schizophrenia patients. This paper systematically reviews the prevalence and treatment of somatic co-morbidity in schizophrenia patients in general practice.

**Methods:**

The MEDLINE, EMBASE, PsycINFO data-bases and the Cochrane Library were searched and original research articles on somatic diseases of schizophrenia patients and their treatment in the primary care setting were selected.

**Results:**

The results of this search show that the incidence of a wide range of diseases, such as diabetes mellitus, the metabolic syndrome, coronary heart diseases, and COPD is significantly higher in schizophrenia patients than in the normal population. The health of schizophrenic patients is less than optimal in several areas, partly due to their inadequate help-seeking behaviour. Current GP management of such patients appears not to take this fact into account. However, when schizophrenic patients seek the GP's help, they value the care provided.

**Conclusion:**

Schizophrenia patients are at risk of undetected somatic co-morbidity. They present physical complaints at a late, more serious stage. GPs should take this into account by adopting proactive behaviour. The development of a set of guidelines with a clear description of the GP's responsibilities would facilitate the desired changes in the management of somatic diseases in these patients.

## Background

Patients suffering from chronic psychotic disorders run an increased risk of developing somatic diseases. These chronic psychotic disorders include schizophrenia, schizoaffective disorders, bipolar disorders and recurrent psychotic depressions. Schizophrenia patients, especially, run a higher risk of disease and early death [1]. Multiple factors contribute to this risk [2-5]. Intrinsic causes are the mental and physical stress accompanying the disorder, as well as the negative symptoms, such as cognitive retardation and loss of initiative. Extrinsic causes include unhealthy life style, smoking, consumption of fast food, lack of exercise and the side effects of pharmacotherapy [6]. Antipsychotic medication also increases the chance of developing metabolic syndrome and diabetes mellitus [7].

Leucht et al. indicated, in their comprehensive review of the literature, four causes for the increased physical co-morbidity in patients with schizophrenia: disease-related factors, drug treatment-related factors, system-related factors including stigmas on mental illnesses, and physician related factors [8].

The majority of schizophrenia patients contact the general practice rather frequently [9], yet they often face delay in diagnosis and treatment of somatic co-morbidity [10]. GPs need to be better informed about the increased risk of somatic co-morbidity in these patients in order to be able to provide adequate health care for them [8]. Additionally, it has been recognised that there is much room for improvement in the collaboration between primary and secondary care [11,12].

Currently, GPs have no guidelines on how to manage somatic morbidity in patients with chronic psychosis. It is supposed that the development and implementation of a set of guidelines would improve the quality of care for schizophrenia patients. An improvement could be achieved if these guidelines addressed the deficiencies in the care of GPs for these patients. Therefore it was decided to collect data on somatic co-morbidity in patients with chronic psychosis and on their treatment in general practice with the objective of determining the need for a specific set of guidelines for GPs.

The following questions were posed:

- What is the prevalence of somatic co-morbidity among schizophrenia patients in general practice?

- Does the primary care provided for somatic diseases meet the needs of these patients?

## Method

A search was made of literature published between 1990 and September 2007 in the medical databases MEDLINE, EMBASE, PsychINFO and the Cochrane Library. Very few articles were found concerning schizophrenia patients in general practice and therefore a wider search was conducted using the key words 'mental disorders' OR 'psychosis' OR 'schizophrenia' AND 'somatic problems' OR 'physical illness' OR 'physical disease' OR 'diabetes mellitus' OR 'cardiovascular disease' AND 'general practice' OR 'general practitioner' OR 'family physician' OR 'family practice'. As a result 186 abstracts were found and from these were selected the original research articles concerning schizophrenia patients from the general population, casu quo the patients in ambulatory care who constitute the population of primary health care. From the bibliographies of these articles, more articles were selected based on their relevance to family health care practice. The study was restricted to articles on health problems that required the GP to decide on treatment policies. Articles on general quality of life were excluded, as were research articles referring to mortality or abnormal laboratory values without health problems. From studies that addressed multiple questions, only those conclusions that were relevant to the two research questions were considered.

Articles were selected by the authors, independently of each other. The two lists of selected articles were compared and, following discussions, consensus was reached as to which articles should be included in the study and which should be rejected. There was disagreement over one article but it was finally excluded as it was not applicable to primary care. A record sheet was developed to present the relevant information from each study accurately.

## Results

A total of 15 original research articles on somatic co-morbidity in patients suffering from psychotic disorders and 6 original research articles concerning diagnostic procedures and treatment of somatic co-morbidity form the basis of this paper.

### Prevalence of co-morbidity (table 1)

**Table 1:** Somatic co-morbidity of schizophrenia patients

*First author*	*Period*	*Design*	*N*	*Results*	*Conclusion*
Jones, 2004 [13]. USA	1996–2000	Cross-sectional comparative study of Medicaid claims	147 pts with SMI (78 schizophr. pts)	**Prevalence**: 74% of pts with SMI were treated for a chronic physical health problem. 50% had two or more chronic physical diseases. Chronic pulmonary disease was most prevalent: 31%	Risk adjustment for physical health is essential when setting performance standards or cost expectations for mental health treatment.
Dixon, 2000[14]. USA	1991–1996	Retrospective analysis of 2 databases and interviews (PORT)	Medicaid 6066; Medicare 14182; PORT 719	**Prevalence**: Schizophr. pts treated for DM: 9–14%. DM in schiz. pts. aged 65+: 18.8–20.8%; DM in schizophr. women: 15%–21.9%; DM in schizophr. black pts: 11.6–18.5%	Before the widespread use of the atypical antipsychotic drugs, diabetes was a major problem for persons with schizophrenia. Being older, female, or black increased the likelihood of DM.
Carlson, 2006[15]. UK	1994–2002	Retrospective cohort study in UK General Practice Research Database	59089 conv. antipsych users; 9053 atyp antipsych users; 1491548 ctrls	**HR **of DM: conv. antipych: 1.9; atyp. antipsych: 2.9	There is an increased risk of developing diabetes during treatment with antipsychotics.
Sacchetti, 2005[16]. Italy	1996–April 2002	Retrospective age- and sex-matched cohort study in a general practitioners database	2071 haloper. 266 olanzap. 567 risperid. 109 quetiapine 6012 ctrls	**HR **of DM conv. antipsych: haloperidol: 12.4; atyp antipsych: olanzapine: 20.4; risperidone: 18.7; quetiapine: 33.7	The incidence of diabetes is significantly higher in patients taking antipsychotics.
Kornegay, 2002[17]. UK	1994–1999	Nested case-control study in UK General Practice Research Database	424 newly diagnosed DM pts vs. 1522 ctrls	**OR **of current antipsych. exposure in pts. with incident DM: 1.7	This study showed an increased risk of incident diabetes among current users of atypical and conventional antipsychotic medication.
Meyer, 2005 [18]. USA	2001–June 2003	Cohort study, using baseline data from CATIE Schizophrenia Trial	1231 schizophr. pts 18–65 years of age	**Prevalence **of metabolic syndrome in schizophr. pts: 35.8%. Pts with metab. syndr. rate themselves lower on physical health.	The metabolic syndrome is highly prevalent in schizophr. pts and is strongly associated with a poor self-rating of physical health and increased somatic preoccupation.
Lamberti, 2006[19]. USA	Not mentioned	Cross-sectional comparative study	93 clozapine users vs. 2701 ctrls	**Prevalence **of metabolic syndrome: clozapine: 53.8%; controls: 20.7%	Patients receiving clozapine are at significantly increased risk for developing the metabolic syndrome.
Osborn, 2006[20]. UK	2003	Cross-sectional screening	74 pts with SMI vs. 48 ctrls	**OR **of raised 10-years CHD risk among patients with SMI: 1.8; **OR **of raised chol/HDL ratio: 1.8. **OR **of DM: 3.8. **OR **of smoking: 3.0	Patients with non-affective chronic psychotic illness have excess risk factors for coronary heart disease, which are not wholly accounted for by medication or socio-economic deprivation.
Samele, 2007[21]. UK	1999–2001	Case-control study of first episode psychosis (FEP) patients	89 FEP pts vs. 89 ctrls matched on age and sex	**OR **of current physical illness: 2.85. **OR **of smoking: 1.82. **OR **of eating fast-food: 1.04	Some risk factors for physical health problems are present at the onset of psychosis, but these may be explained by unemployment.
Himelhoch, 2004[22]. USA	Mrt 2000–Dec 2000	Cross- sectional comparative survey	185 SMI pts vs. 2706 ctrls matched on age, gender, and race	**Prevalence **of: current smoking 60,5%, COPD 22.6%, astma 18.5%	Prevalence of COPD is significantly higher among patients with SMI. Predictive factors were age, being male, and being a current smoker.
Carney, 2005[23]. USA	1996–2002	Retrospective analysis of longitudinal claims data	1074 pts with schizophr. or schizoaffective disorder vs. 726262 ctrls	**OR **of: COPD 1.88, complicated DM 2.11, hypothyreoidism 2.62, hepatitis C 7.54, electrolytdisorders 4.21	Schizophrenia is associated with substantial chronic medical burden. Familiarity with conditions affecting schizophr. pts may assist programs aimed at providing medical care for the mentally ill.
Lichtermann, 2001[24]. Finland	1971–1996	Cohort study	26.996 schizophr. pts, 39131 parents, 52976 siblings	**SIR **of: cancer incidence 1.17, lung cancer 2.17, pharynx cancer 2.60	Schizophr. pts have an increased risk of pharynx- and lung cancer. This may be the consequence from lifestyle factors, particularly tobacco smoking and alcohol consumption.
Hippisley-Cox, 2007 [25]. UK	1995–2005	Population-based nested case-control study	139 schizophr. pts vs. 571 ctrls	**OR **of mamma ca 1.52, colon ca 2.90, respiratory ca 0.53	Schizophr. pts have a higher risk of colon cancer and a lower risk of respiratory cancer compared with controls after adjustment for confounders.
Kuritzky, 1999[26]. Israël	1999	Cross-sectional comparative survey	108 schizophr. pts vs. 100 ctrls	**Prevalence **of headache in pts: 48%; headache in ctrls: 41%. No sign. difference on comparing the type of headache between groups.	Schizophr. pts describe the same type, frequency, severity and duration of headache compared with controls, but tend to refrain from complaining about their headache.
Viertiö, 2007[27]. Finland	Sept 2000–June 2001	Cross-sectional comparative study	Distance VA measured: 56 schizophr. pts vs. 6588 ctrls. Near VA measured: 51 schizophr. pts vs. 6415 ctrls	**OR **of schizophr. pts with visual impairment for distance: 5.04 and for near vision: 6.22 **Prevalence **of schizophr. pts having visual examination during previous 5 years: 43.9%. vs. total sample: 69.7%	Schizophr. pts attend visual examinations less frequently than others, and their vision is notably weaker. Regular ocular evaluations should be included in physical health monitoring.

HR = Hazard Ratio

OR = Odds Ratio

SIR = standardized incidence ratio

DM = diabetes mellitus

SMI = severe mental illness

VA = visual acuity

Research articles on somatic co-morbidity comprised 6 database studies, 5 cross-sectional studies, one cohort study, two nested case-control studies, and one case-control study. The database studies included large retrospective studies from 4 general practitioners' databases: 3 from the UK and 1 from Italy, 1 national database from Finland's National Hospital Discharge and Disability Pension registers, and 3 databases from social insurance organisations in the USA. The research data from Meyer et al came from the Clinical Antipsychotic Trials of Intervention Effectiveness (CATIE) study base file. It was decided to include this study since the data came from a cohort of non-admitted schizophrenic patients.

The prevalence of chronic physical illnesses among patients with severe mental illness (SMI) was found to be high. Jones et al. found it to be as high as 74% in a small cross-sectional comparative study among 147 Medicaid enrolled patients (mean age ± SD of 38 ± 10 years) with severe mental illness [13]. In this study chronic pulmonary illness was the most prevalent (31% incidence). However, this diagnosis was more frequently given to patients with an affective psychosis than to schizophrenia patients. Infectious diseases caused the highest average annual costs of treatment, and were often associated with substance abuse and homelessness. Age, obesity, and substance abuse were predictive of health problem severity.

#### Diabetes mellitus

Diabetes mellitus was the first chronic disease to be recognised in schizophrenic patients and in patients who use antipsychotic drugs. Also, a much higher prevalence of diabetes mellitus (9–14%) was found in 5 studies, from three different countries, of which the study of Kornegay et al. was prospective, and the other 4 retrospective [13-17]. The relative risk of developing diabetes mellitus is 2–3 times higher in schizophrenic patients than in non-schizophrenic. Dixon et al. carried out a study using two large insurance databases in the USA, Medicaid and Medicare, and also interviewed 719 schizophrenic patients in two states. The patients had been diagnosed with schizophrenia, including both schizoaffective and schizophreniform disorders. The prevalence of current treated diabetes varied from 9 to 14 percent. Being older, female, and African-American was associated with an increased likelihood of diabetes. Dixon's study, in the early 1990s, suggests that even before the widespread use of atypical antipsychotic drugs, diabetes was a major problem for persons with schizophrenia [14].

Carlson et al. reports the results of a retrospective cohort study to determine the incidence of diabetes mellitus in patients exposed to conventional or atypical antipsychotic drugs compared to a general practice population in the UK General Practice Research Database. The incidences of diabetes during exposure to conventional antipsychotic drugs was 7.7 per 1000 patient-years (CI = 6.7–8.7) and 9.8 per 1000 patient-years (CI = 7.4–12.2) during exposure to atypical antipsychotic drugs. This is significantly higher than the incidence of diabetes in the patient population of the General Practice Research Database, which was 3.3 [15].

Sacchetti et al. also carried out a retrospective cohort study in an Italian general practice database [16]. They compared subjects who were exposed and not exposed to antipsychotic drugs. They compared the incidence of diabetes (per 1000 person-years) in patients taking haloperidol (N = 2071), olanzapine (N = 266), risperidone (N = 567) and quetiapine (N = 109) with a control group (N = 6026). The ratios found were: 12.4% for the haloperidol group, 20.4% for the olanzapine group, 18.7% for the risperidone group, and 33.7% for the quetiapine group. The four treatment groups differed too much in size to draw specific conclusions about each single drug.

Kornegay et al. carried out a nested case-control study in UK General Practice Research Database among adults prescribed at least one course of treatment with an antipsychotic drug between January 1994 and December 1998 and compared them with age, gender and practice matched controls [17]. The results showed elevated risk of incident diabetes (Odds ratio 1.7) associated with current exposure to atypical or conventional antipsychotic drugs, independent of the risk due to other established risk factors.

#### Cardiovascular risk factors

Schizophrenia patients have a higher chance (prevalence of 36%) of developing metabolic syndrome, even without antipsychotic medication. This is generally accompanied by a poor self-experienced physical health [18]. Lamberti et al. conducted a cross-sectional comparative study which showed an additional increased risk of 53.8% for metabolic syndrome among users of clozapine, versus 20.7% in the control [19]. Not only diabetes mellitus but multiple risk factors for cardiovascular diseases are significantly increased in this patient group [20]. Schizophrenic patients have a higher risk of raised cholesterol/HDL ratio, and also smoke more often.

Some risk factors are already present at the onset of the psychotic disorder. Samele et al. compared eighty-nine patients with a first episode psychosis (FEP) to age- and sex matched controls for self-reported physical illness and health risk factors [21]. Patients with a first episode psychosis were more likely to be cigarette smokers and eat fast food, although these risk factors may be explained by unemployment.

#### Respiratory system

Respiratory problems, COPD and a deteriorated lung capacity occur significantly more frequently [21-23]. Himelhoch et al. interviewed a sample of SMI patients (60% were current smokers, mean age 44 years), and compared the results to a matched subset of national comparison subjects. The self-reported prevalence of COPD among schizophrenic patients was 22.6%. Carney et al analysed longitudinal administrative claim data of schizophrenia patients and controls and found an OR of 1.88 for COPD [23].

#### Other co-morbidity

Besides COPD, Carney et al. found in their large database study an increased risk of the following conditions: hypothyroidism, hepatitis C and electrolyte disorders [23]. Lichtermann et al. found an increased risk of pharynx- and lung cancer in their large-scale Finnish database study [24], but Hippisley-Cox et al. found in their small case-control study a lower risk of respiratory cancer, and a higher risk of mamma cancer and colon cancer [25]. When asked, schizophrenic patients appear to suffer from migraines and tension headaches as much as the control group [26]. However, they did not report this spontaneously and they usually sought help quite late. This is also shown by the fact that schizophrenia patients had much more deteriorated vision both for distance and for near vision, because they were less likely to visit an ophthalmologist or optician when their eyesight was impaired [27].

### Screening, diagnostic procedures and treatment of somatic co-morbidity (table 2)

**Table 2:** Screening, diagnostic procedures and treatment of somatic co-morbidity

*First author*	*Period*	*Design*	*N*	*Results*	*Conclusion*
Tsay, 2007 [26]. Taiwan	1997–2001	Cohort study	97589 pts admitted for acute appendicitis	**OR **of perf. appendix: no mental disorder: 1.0; schizophr. pts: 2.83; affective psychosis: 1.15; other mental disorders: 1.58	Mentally ill patients are at a disadvantage in obtaining timely treatment for their physical diseases. Schizophrenic pts are the most vulnerable ones for obtaining timely surgical care.
Nasrallah, 2006[27]. USA	2003	Cohort study, baseline data from CATIE Schizophrenia Trial	1460 schizophr. pts 18–65 years of age	**Prevalence **of pts with untreated DM: 30.2%; pts with untreated hypertension: 62.4%; pts with untreated dyslipidemia: 88.0%	There is a high likelihood that metabolic disorders are untreated in schizophr. pts.
Roberts, 2007[28]. UK	April 1998–Dec 2000	Case-matched retrospective review.	195 schizophr. pts vs. 390 matched asthma pts vs. 390 matched controls	**OR **of blood pressure records 0.51 (vs. asthma pts); **OR **of cholesterol records 0.50 (vs. asthma pts); **OR **of blood pressure records 0.68 (vs. controls); **OR **of cholesterol records 0.58 (vs. controls)	Schizophr. pts are less likely to receive some important general health checks than patients without schizophrenia.
Wright, 2006[29]. UK	Not mentioned	Qualitative research	31 SMI pts, 8 GP's and 2 NP's, 25 mental health workers	Identified problems are the lack of familiarity with SMI and antipsychotic side effects in general practice, poor communication of physical health issues to the CMHT, lack of knowledge regarding CHD risk factor screening, and difficulties in interpreting screening results and implementing appropriate interventions in secondary care	Management of physical health care for people with SMI requires complex solutions that cross the primary-secondary care interface.
Osborn, 2003[30]. UK	Not mentioned	Experiment	182 psychotic pts 313 controls	**OR **for pts participating in cardiovascular risk screening: 0.76. Psychotic pts consulted their GP more often (mean difference 1.8)	Interest in risk assessment was similar to those in other community research involving blood tests.
Beecroft, 2001[31]. UK	Not mentioned	Interviews	309 randomly selected pts from a sample of 566 psychotic pts	Pts who visited their GP within the last 6 months were more often (83% vs. 50%) satisfied with the amount and type of service provided for their physical needs	Patients with SMI should be encouraged to see their GPs. There is a strong argument for a routine annual check up of the severely mentally ill by their GPs.

OR = Odds ratio

SMI = severe mental illness

CMHT = community mental health teams

CHD = coronary heart disease

Two cohort studies and one case-matched retrospective review dealt with the level of health-care provision for psychotic patients, and three studies examined patients' views on diagnostic procedures and physical health care.

Tsay et al. conducted research among a large number of acute patients with appendicitis [28]. They showed that schizophrenia patients had a 2.8 increased chance of a ruptured appendix at the start of the treatment. In addition, in schizophrenia patients chronic diseases like hypertension, dyslipidaemia and DM were under-diagnosed and under-treated (30–88%) [29]. Roberts et al. reviewed case notes of 195 schizophrenia patients and 390 matched controls [30], and found that schizophrenia patients were significantly less likely to have had their blood pressure or cholesterol recorded. GPs often appeared not to be aware of the risk of somatic co-morbidity in schizophrenia patients, and they knew little of the side-effects of antipsychotic medication [31].

Osborn et al. invited 182 schizophrenia patients and 313 controls for a cardiovascular risk assessment in general practice. The interest in risk assessment among the psychotic patients was higher than the researchers expected, but lower than the control group: odds ratio 0.76 (0.53–1.10) [32]. The psychosis group consulted their GPs more often – mean difference 1.8 (0.8–2.9) per year.

Beecroft et al. investigated whether SMI patients received better health treatment if they attended their GP, a Community Mental Health Team, or both. They interviewed 309 patients with psychotic disorders. Patients who had seen their GP in the previous 6 months were more likely to be satisfied with the service provided for their physical health. They suggested that the health needs of schizophrenic patients might be better met if the GP adopted a more proactive follow-up policy which encouraged the patient to see their GP who was responsible for physical health service provision [33].

## Discussion

The data show that the prevalence of somatic morbidity in patients with chronic psychosis is indeed considerably higher than in an open population [34]. This somatic morbidity concerns a scala of diseases ranging from diabetes mellitus to hepatitis C.

Patients with chronic psychosis are at risk of under-diagnosis and under-treatment. The nature of their mental illness makes it difficult for these patients to interpret body signals correctly. Moreover, schizophrenia patients are not inclined to discuss complaints spontaneously and find it difficult to call for help, while some patients do not like others to interfere [35]. Both chronic and acute conditions surface at a late stage. In addition to this, the illness is accompanied by negative symptoms and cognitive deterioration, which lead to inactivity and loss of initiative. This contributes to higher risks of poor attendance and self care. Schizophrenic patients have trouble adjusting to society's demands, which causes stress. In order to cope with this stress and the psychotic symptoms, they take refuge in nicotine, cannabis, alcohol and other narcotics. Unfortunately, the use of substances to self-medicate may exacerbate the psychotic symptoms, thus creating a vicious cycle, which leads to an existence on the edge of society.

The above-mentioned limitations make these patients less inclined to request medical assistance [35,36]. As a consequence, the health of schizophrenic patients suffers in several areas. However, when schizophrenic patients seek GP's assistance, the care provided is highly valued [37,38]. Patients who visit the GP regularly are often satisfied with the care provided and their health needs may be better met if the GP applied a more proactive follow-up policy which encouraged the patients to see their GP [32,33].

Patients with severe mental disease, suffer from a loss of perspective and loss of hope. These are patient-related factors which might play a role in their appreciation of diagnostic procedures. No evidence of any research has been found that explores the role of these factors in the tacit understanding between the GP and his patient.

Compassion and offering an easily-accessible care for patients are priorities for GPs [39]. They know from experience that coming too close to a psychotic or paranoid patient can result in his withdrawal from medical care. The GP needs to balance between offering the necessary care without losing contact with the patient.

### Limitations

A few studies dealt with patients suffering from 'severe mental illness' (SMI); the SMI label comprises a heterogenic group that largely consists of psychotic patients. Four studies concerned the somatic co-morbidity in antipsychotic drugs users, and three studies examined insurance claims data.

Most of the studies that were included came from the United Kingdom (n = 9) and the United States (n = 7); two articles came from Finland, one from Italy, one from Israel, and one from Taiwan. In the health care system of these countries the GPs provide all primary health care, although the position and functions of GPs might be somewhat different. Our results cannot be generalized to countries with other healthcare systems.

The methodology of the studies enclosed varied markedly, thus resulting in evidence of varying strength. The studies of Kornegay, Samele and Hippisley-Cox, being case-control studies, provide the best quality of evidence with regard to the first research question, while Roberts' study does so for the second research question. Although the figures of the enclosed studies vary, it is emphasized that the outcomes do not contradict each other, but all come to the same conclusions with one exception: the risk of lung cancer. The results of the enclosed studies were presented in different outcome measures, e.g. odd ratios, hazard ratios, Cox proportional hazard analysis, standardized incidence ratios and prevalence statistics (percentages). This made it impossible to calculate 'overall' numbers.

Because it was felt necessary to add the search terms 'diabetes mellitus' and 'cardiovascular disease' to the search, it is possible that these topics are overrepresented in our study.

## Conclusion

In the enclosed studies, patients suffering from schizophrenia and related psychoses run a substantial risk of developing diabetes mellitus, metabolic syndrome, hypertension, cardiovascular diseases, lung diseases such as COPD, hypothyroidism and visual problems. The health of these patients is less than optimal in several areas, due to disease-related factors, to drug treatment-related factors, to patient-related factors, and to physician-related factors.

GPs should be aware of the high risk of somatic co-morbidity in this specific patient group and take into account their cognitive and social handicaps by being alert and proactive when diagnosing. While at the same time, however, GPs should also inquire after and respect the patient's value judgement concerning screening, diagnostic procedures, and treatment.

The development of a set of guidelines for GPs, giving a clear description of responsibilities with regard to somatic co-morbidity, would facilitate the necessary change in the GP's management of and contribute to an improvement in primary care for these patients.

## Competing interests

The authors declare that they have no competing interests.

## Authors' contributions

MO and BM independently reviewed the extracted data, and summarized the included studies. MO drafted the manuscript. Both authors contributed to the writing of the final paper.

## Pre-publication history

The pre-publication history for this paper can be accessed here:


